# Effects of Ginger Supplementation on Markers of Inflammation and Functional Capacity in Individuals with Mild to Moderate Joint Pain †

**DOI:** 10.3390/nu17142365

**Published:** 2025-07-18

**Authors:** Jacob Broeckel, Landry Estes, Megan Leonard, Broderick L. Dickerson, Drew E. Gonzalez, Martin Purpura, Ralf Jäger, Ryan J. Sowinski, Christopher J. Rasmussen, Richard B. Kreider

**Affiliations:** 1 Exercise & Sport Nutrition Laboratory, Human Clinical Research Facility, Department of Kinesiology and Sports Management, Texas A&M University, College Station, TX 77843, USA; broeckelj@tamu.edu (J.B.); landry.estes@tamu.edu (L.E.); meganleonard10@tamu.edu (M.L.); dickersobl5@email.tamu.edu (B.L.D.); dg18@tamu.edu (D.E.G.); rjs370@tamu.edu (R.J.S.);; 2 Increnovo LLC, Whitefish Bay, WI 53217, USA; martin.purpura@increnovo.com (M.P.); ralf.jaeger@increnovo.com (R.J.)

**Keywords:** knee, hip osteoarthritis, muscle soreness, dietary supplement, analgesic, quality of life

## Abstract

**Background:** Ginger contains gingerols, shagaols, paradols, gingerdiones, and terpenes, which have been shown to display anti-inflammatory properties and inhibit pain receptors. For this reason, ginger has been marketed as a natural analgesic. This study examined whether a specialized ginger extract obtained through supercritical CO_2_ extraction and subsequent fermentation affects pain perception, functional capacity, and markers of inflammation. **Methods:** Thirty men and women (56.0 ± 9.0 years, 164.4 ± 14 cm, 86.5 ± 20.9 kg, 31.0 ± 7.5 kg/m^2^) with a history of mild to severe joint and muscle pain as well as inflammation participated in a placebo-controlled, randomized, parallel-arm study. Participants donated fasting blood, completed questionnaires, rated pain in the thighs to standardized pressure, and then completed squats/deep knee bends, while holding 30% of body mass, for 3 sets of 10 repetitions on days 0, 30, and 56 of supplementation. Participants repeated tests after 2 days of recovery following each testing session. Participants were matched by demographics and randomized to ingest 125 mg/d of a placebo or ginger (standardized to contain 10% total gingerols and no more than 3% total shogaols) for 58 days. Data were analyzed by a general linear model (GLM) analysis of variance with repeated measures, mean changes from the baseline with 95% confidence intervals, and chi-squared analysis. **Results:** There was evidence that ginger supplementation attenuated perceptions of muscle pain in the vastus medialis; improved ratings of pain, stiffness, and functional capacity; and affected several inflammatory markers (e.g., IL-6, INF-ϒ, TNF-α, and C-Reactive Protein concentrations), particularly following two days of recovery from resistance exercise. There was also evidence that ginger supplementation increased eosinophils and was associated with less frequent but not significantly different use of over-the-counter analgesics. **Conclusions:** Ginger supplementation (125 mg/d, providing 12.5 mg/d of gingerols) appears to have some favorable effects on perceptions of pain, functional capacity, and inflammatory markers in men and women experiencing mild to moderate muscle and joint pain. Registered clinical trial #ISRCTN74292348.

## 1. Introduction

Ginger (*Zingiber officinale* Roscoe) is a flowering plant found primarily in Southeast Asia that has been utilized as a spice and in ayurvedic medicinal practices. It is typically consumed as a whole root, in extract powders, and as herbal tea [1]. Eastern medicine has utilized ginger to address various ailments, including cold, nausea, fever, headache, and gastrointestinal issues [2]. Ginger has been shown to be neuroprotective, gastroprotective, and modulate inflammation due to its pharmacological activity [3]. The anti-inflammatory effects of ginger have been attributed to its phenolic compounds (e.g., gingerols, shogaols, paradols, and zingerone) [1,4,5,6]. In its raw form, gingerols are the primary polyphenols found within the ginger rhizome [6]. Gingerol compounds are the most studied aspects in terms of anti-inflammatory, antioxidant, and anticancer effects [1,7,8,9,10,11]. However, evidence is emerging that other ginger-derived phenols have similar benefits [9,12]. Different processing methods transform gingerols into shogaols, paradols, and zingerone due to slight alterations in the molecular structures of these compounds. Shogaols and zingerone are produced when gingerol receives heat treatment through roasting, cooking, or dehydration. Hydrogenation reduces the double bond in shogaols, producing paradols [1]. While these compounds display unique properties, stability, and pharmacological activity, they have all been shown to help mediate inflammation and oxidative stress.

Ginger-derived phenols have been reported to provide anti-inflammatory effects by inhibiting macrophage and neutrophil activation, decreasing the pro-inflammatory cytokines that upregulate cyclooxygenase enzyme-2 isoenzyme (COX-2) expression, and decreasing Nuclear Factor-kappa β (NF-κβ) and Protein Kinase B (Akt) that promote cytokine gene expression [1,7,13,14]. Over-the-counter (OTC) nonsteroidal anti-inflammatory drugs (NSAIDs) are often taken for reducing inflammation [15,16]. They primarily act to inhibit the COX enzyme, including the COX-2 isoenzyme, which promotes inflammation [1,2], and the COX-1 isoenzyme, which affects the intestinal mucosa lining, kidneys, and platelet aggregation [16]. Ginger has also been reported to inhibit the vanilloid subtype 1 (TRPV1) pain-sensitive receptor [17,18,19,20]. Although NSAIDs are FDA-approved, chronic use may negatively affect the kidney, liver, and gastrointestinal mucosa, and/or promote adverse cardiovascular events [21,22]. As such, finding natural alternatives to NSAIDs that may reduce inflammation and/or perceptions of pain with fewer side effects, like ginger, is of particular interest [1,16,17,23,24,25,26,27,28,29,30,31,32,33]. However, the species and even cultivar of ginger can affect the strength and efficacy of its analgesic effects, primarily due to differences in phytochemical composition, especially the concentrations of active compounds like [6]-gingerol, [6]-shogaol, [8]-gingerol, and [10]-gingerol. For this reason, methods with which to extract these compounds from ginger species have been developed to standardize potency. Most sources of ginger only provide 1–2% gingerol, requiring the ingestion of 1–2 g of ginger powder to provide 10–30 mg of gingerols recommended for therapeutic benefit (e.g., dysmnorrhea, osteoarthritis, muscle soreness, and migraine/headache), with a minimum effective dose of about 10 mg of gingerols [9,34,35,36]. A high-potency specialized ginger extract has recently been marketed as an effective source of ginger, where the gingerols and other bioactive compounds found primarily in the ginger oleoresin are concentrated through a CO_2_ extraction, fermentation, and drying process. Extracting gingerols from oleoresin has been reported to improve absorption and bioavailability [37,38]. Theoretically, it would only take about 125–150 mg of this source of ginger to provide therapeutic benefit, thereby making it much more practical to use it as a dietary ingredient. However, research is needed to determine if this source and dosage of ginger are efficacious.

This study examined the effects of supplementing the diet with a higher-potency ginger extract on perceptions of pain and markers of inflammation in individuals who experience mild joint pain in response to physical activity. The primary outcomes were ratings of muscle pain, functional capacity questionnaires, and markers of inflammation. Secondary outcomes included joint flexibility, quality of life, use of over-the-counter analgesics, clinical blood markers of safety, and reported side effects. Given the known anti-inflammatory properties of ginger, we hypothesized that supplementation with this source of ginger would reduce perceptions of pain and markers of inflammation.

## 2. Methods

### 2.1. Research Design

This study was conducted as a randomized, placebo-controlled, double-blind, parallel-group, and repeated-measures study at a university clinical research facility. The Human Protection Institutional Review Board approved this study (IRB2022-1345, approved on 5 April 2023) in agreement with the Declaration of Helsinki human research ethics. Each participant signed written consent forms for participation in this investigation. This study was registered by an International Standard Randomized Controlled Trial Number (ISRCTN#74292348, submitted on 30 April 2025, posted on 7 May 2025). The independent variable was dietary supplementation. The primary outcomes were subjective ratings of muscle pain, functional capacity questionnaires, and markers of inflammation. Secondary outcomes included range of motion and flexibility, perceptions of quality of life, clinical chemistry panels, the frequency and quantity of use of OTC analgesics, and reported side effects.

### 2.2. Study Participants

The recruitment of participants was conducted through email, social media posts, and study flyers in online and print formats. Potential participants were contacted to determine their eligibility. Inclusion criteria included the following: (1.) healthy males and females 40 to 75 years of age; (2.) a history of mild to severe joint and muscle pain with evidence of elevated inflammatory markers in the blood upon entry and/or a history of physician-diagnosed osteoarthritis; and (3.) medically stable with no current uncontrolled cardiovascular, metabolic, or pulmonary disease. Participation was granted if participants were taking medications that would not affect study outcomes for non-related chronic diseases or disorders (e.g., to manage blood pressure, blood lipids, thyroid conditions, blood glucose, and low-dose aspirin).

Exclusion criteria included (1.) no history of mild to severe joint and/or muscle pain; (2.) a history of uncontrolled cardiovascular, metabolic, or pulmonary disease; (3.) pregnancy or an aspiration to become pregnant during the investigation; (4.) current use of prescription COX-2 inhibitor medications, corticosteroids, or disease-modifying antirheumatic drugs (DMARDS), Interleukin-1 inhibitors, T-cell inhibitors, B-cell inhibitors, or Janus kinase inhibitors; (5.) a history in the prior month of bleeding disorders or the current use of prescription blood thinner medications; (6.) inability to perform functional exercise tasks to be used in the investigation; and (7.) no known allergies to ginger or Gum Arabic. All participants provided voluntary and written informed consent to participate in this study in compliance with IRB standards.

**Figure 1:**
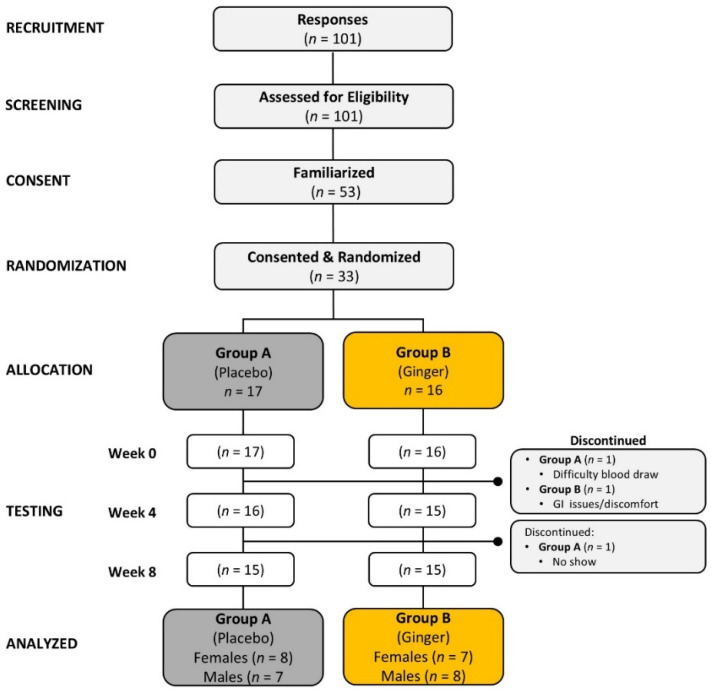
Consolidated Standards of Reporting Trials (CONSORT) flow chart for recruitment, randomization, allocation, completion, testing sessions, and analysis of the treatment groups. *n* = sample size.

Figure 1 shows a Consolidated Standards of Reporting Trials (CONSORT) diagram. One hundred and one (101) individuals responded to recruitment efforts and were screened for eligibility. A total of 53 individuals met preliminary screening eligibility requirements and proceeded to familiarization sessions. Thirty-three individuals were randomized after consenting to the investigation and counterbalanced into one of two treatment conditions. The number of participants randomized to each treatment and completing each session is displayed in Figure 1. Two participants discontinued the investigation due to difficulty in obtaining blood samples (*n* = 1, placebo) and self-reported gastrointestinal issues resulting from supplement ingestion (*n* = 1, ginger). One participant became absent and withdrew after completing their first five testing sessions (placebo). Therefore, 30 participants (15 females and 15 males) completed the study, with 15 participants in each group (placebo: 8 females, 7 males; ginger: 7 females, 8 males). Statistical analysis was performed on 30 participants who completed the study.

### 2.3. Familiarization

**Figure 2:**
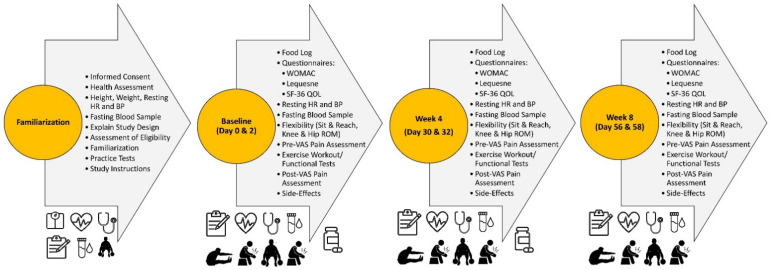
Overview of experiment study design. HR = resting heart rate, BP = blood pressure, WOMAC = Western Ontario and McMaster’s University Osteoarthritis Index, QOL = quality of life, ROM = range of motion, and VAS = visual analog scale.

Figure 2 displays the experimental approach and testing sequence. An invitation to a familiarization session was extended to individuals who met preliminary eligibility criteria. During this session, information on the study protocols was provided, with completion of a written informed consent to follow. Those who met the inclusion criteria completed a health assessment that included the measurement of height, weight, resting heart rate (HR), and blood pressure (BP), and the donation of a fasted (≥12 h) blood sample, which was used for screening to assess eligibility. Participants were familiarized and allowed to practice the functional exercise tests (e.g., 3 sets × 10 repetitions of deep knee bends with dumbbells approximating 30% of body mass). Finally, they were provided with instructions on completing food logs. Participants were directed to document detailed food and energy-containing fluid intake for 3 weekdays and 1 weekend day before the second, fourth, and sixth visits to the lab. They were instructed to fast for 12 h, refrain from intense exercise for 24 h, and refrain from OTC analgesic use for 48 h.

### 2.4. Experimental Session Testing Protocol

Following the familiarization session, participants completed a baseline testing session and two follow-up testing sessions, each with a 48 h follow-up testing session (six sessions total). These baseline sessions were separated by approximately 4 weeks. Participants reported to the lab and returned 4-day food logs during all baseline sessions. They were then asked to complete quality of life and perception of pain questionnaires, including the WOMAC Osteoarthritis Global Index, the Lequesne Functional Index, and the SF-36 Quality of Life (QOL) Inventory. Resting HR and BP were obtained, followed by the procurement of a venous blood sample. Range of motion (ROM) measurements were then conducted using unassisted knee and hip flexion and measured with a 12″ goniometer. This was followed by performing a sit-and-reach test to assess flexibility. A visual analog scale (VAS) was used to assess perceptions of pain in response to standardized pressure applied to several areas of the thigh before and after performing the functional exercise test (described below). Participants then reported to the lab 48 h later to conduct all measurements without the incorporation of the exercise intervention (Figure 2). Participants repeated this two-day experimental session after 4 and 8 weeks of intervention.

### 2.5. Functional Exercise Intervention

Participants performed a functional exercise test during each testing session to induce muscle damage and/or soreness. This involved having participants participate in a warm-up at a self-selected pace on a stationary exercise bike for about 5 min prior to performing three sets of ten repetitions of a weighted standing knee bend or squat to normal chair height. Participants held dumbbells of equal weight in each hand, totaling approximately 30% of their body weight. Participants were instructed to squat as deeply as their range of motion would allow. Following each set, a 60 s recovery period was provided.

### 2.6. Supplementation Intervention

Participants were assigned to consume 125 mg/d of a Gum Arabic placebo (Nexira Food, Rouen, France, Lot #190 057) or Organic Ginger Extract Powder (GingerT3^TM^, Specnova LLC, Tysons Corner, VA, USA) standardized to 10% total gingerols and less than 3% shogaols. Supplements were encapsulated by the sponsor and provided as color-matched, tasteless, capsules. The content of the study materials was confirmed by an independent third-party laboratory (Eurofins Analytical Services India Private Limited, Bengaluru, Karnataka, India, AR-24-IR-038509-03, 3 May 2024). Supplements were distributed to participants in pre-blinded boxes, labeled “A” or “B” for double-blind administration. Participants were allocated into one of the two supplemental conditions based on age, gender, and body mass. Participants were instructed to consume the supplements once daily after breakfast or at approximately 8:00 A.M. following the first baseline session (day 0) and continue each day throughout the study or for approximately 58 days. Compliance with supplementation was obtained via online and verbal communication with participants.

### 2.7. Diet Standardization

Prior to the first testing session, participants were directed to document all energy intake including food and fluids for four days before testing and during the 2-day recovery phase. Additionally, they were asked to not partake in intense exercise and OTC analgesic use (e.g., Advil/ibuprofen, Aleve/naproxen, Tylenol/acetaminophen, and aspirin) for 48 h in advance of each testing session and to report to the laboratory after a 12 h fast. Participants were asked to replicate this pattern of eating prior to and following each testing session to standardize caloric intake between testing sessions. Participants recorded dietary intake prior to and during each testing session to assess compliance.

## 3. Procedures

### 3.1. Anthropometrics and Hemodynamics

Height and weight were obtained using a Health-O-Meter Professional 500 KL scale (Pelstar LLC, Alsip, IL, USA). Resting HR and BP were obtained after sitting passively for 5 min. Resting HR was determined by the palpation of the radial artery according to standard procedures. Resting heart rate and blood pressure were measured in the supine position by using a digital heart rate monitor and blood pressure cuff (Connex^®^ ProBP™ 3400; Welch Allyn, Tilburg, NL, USA) [39].

### 3.2. Blood Sampling

Participants provided a fasting venous blood sample (~20 milliliters) at each testing session. Fasting blood was collected into BD Vacutainer^®^ serum separation tubes (SST, Becton, Dickinson and Company, Franklin Lakes, NJ, USA). Samples sat at room temperature (15 min) and were then centrifuged at 3500 rpm for 10 min by using a Thermo Scientific Heraeus MegaFuge 40 R Centrifuge (Thermo Electron North America LLC, West Palm Beach, FL, USA). Serum was aliquoted into microcentrifuge tubes and stored at −80 °C for subsequent analysis. Whole blood was collected into K2 ethylenediaminetetraacetic acid (EDTA) tubes (BD Vacutainer^®^, Becton, Dickinson and Company, Franklin Lakes, NJ, USA). The SST and EDTA samples were sent to the Clinical Pathology Laboratory (Austin, TX, USA) to assess cell blood counts and serum clinical blood profiles. Cytokines were measured from stored samples by using a Cytokine Human Magnetic 10-plex Panel kit with a Luminex™ 200™ Instrument System (ThermoFisher Scientific, Vienna, Austria) using xPONENT^TM^ version 3.1 software. Inter-assay and intra-assay coefficients of variation (CVs) ranged between 2% and 18% and 3% and 10%, respectively. Outliers were identified by using the Grubbs procedure [40]. Platelet aggregation was determined from whole-blood samples by using a CHRONO-LOG^®^ Model 700 Whole Blood/Optical Lumi-Aggregometer with AGGRO/LINK^®^8 and vW Cofactor Software (Havertown, PA, USA, http://www.chronolog.com/Model700.html, accessed on 14 July 2025) according to manufacturer specifications.

### 3.3. Range of Motion and Flexibility Assessment

The determination of knee and hip ROM was conducted by utilizing a standard 12″ goniometer (Medline, Northfield, IL, USA), measuring the flexion of the knee and hip. During the initial baseline session, participants indicated which leg caused the most pain or discomfort. Measurements were repeated on this leg for all of the following testing sessions. Participants were assessed in the supine position with one leg fully extended and the other bent with the foot flat and heel on the table. Flexion ROM was measured on this non-extended leg by participants raising this leg slightly off the table and actively bringing the heel toward the buttocks. While remaining in the supine position, participants were then instructed to passively bring their quadriceps as close to their torso as possible with their leg still in flexion. Investigators measured the flexion ROM at the hip joint. Flexibility was determined using a Sit N’ Reach Trunk Flexibility Box (Figure Finder Flex-Tester, Novel Products, Inc., Rockton, IL, USA).

### 3.4. Pain Assessment

Perceived muscle soreness was evaluated by applying pressure (50 N) to three locations on the quadriceps by using a Commander Algometer (JTECH Medical, Salt Lake City, UT, USA). The three sites were the vastus lateralis (VL) at one-fourth and halfway between the superior border of the patella and the greater trochanter and the vastus medialis (VM) perpendicular to the VL location. This procedure was conducted before and immediately following exercise on baseline testing sessions and once during all 48 h follow up sessions. Participants recorded perceptions of muscle soreness using a VAS ranging from 0 (no pain), 1 (dull ache), 2 (slight pain), 3 (more than slight pain), 4 (painful), 5 (very painful), and 6 (unbearable pain) [41]. Force application followed a standardized order across all sessions: VM, lower VL, and upper VL. Participants were given 3 s to record soreness ratings between tests. Additionally, the Western Ontario and McMaster University Osteoarthritis Index (WOMAC™ 3.1 Index), a 24-question battery, was utilized to quantify facets of pain, joint stiffness, and disability in knee and hip osteoarthritis [42]. The Lequesne Index of Severity of Hip Osteoarthritis 11-item questionnaire was utilized to assess subject ratings of severity of hip osteoarthritis [43,44,45]. Finally, an OTC Analgesic Medication Log was used to record a participant’s analgesic use, frequency of use, and quantity of use throughout the entire study period. Participants completed OTC forms during all 48 h follow-up visits.

### 3.5. Quality of Life

The Short Form Health Survey version 2 (SF-36v2, Rand Health Care, Santa Monica, CA, USA) was administered to evaluate participants’ subjective perceptions of QOL [46]. This survey contains general QOL questions encompassing multiple dimensions of physical and psychological health. This inventory has a test–retest reliability of r = 0.81–0.95 for all domains [47,48].

### 3.6. Functional Capacity

At each testing session, the amount of weight lifted and the number of repetitions performed during each set were recorded. If a participant was unable to complete a set of 10 repetitions, the amount of weight was reduced to complete three sets of ten repetitions. The weight utilized for each set was summed to determine a total lifting volume.

### 3.7. Side Effects

Side effects were evaluated using a frequency and severity of subjective side effects survey, as previously described [49,50,51]. The C_V_ of questions ranged from 1% to 3%, with intraclass correlations between 0.6 and 0.88 [49,50,51]. Participants were also asked to record the use of rescue doses of OTC analgesic medications so that the frequency of use could be assessed.

### 3.8. Statistical Analysis

We performed a comprehensive and statistical analysis [52,53,54] and clinical assessment [52,53,54,55,56,57,58] using methods previously described in detail [59] by using International Business Machines (IBM) Statistical Package for the Social Sciences (SPSS) version 29 statistical analysis software (IBM Corp., Armonk, NY, USA). Numerical data were analyzed using a mixed model general linear model (GLM) analysis of variance (ANOVA) with repeated measures. Mauchly’s test was used to assess sphericity and the kurtosis statistic was used to assess normality. Wilks’ Lambda and Greenhouse–Geisser univariate correction tests were used to adjust for F-value inflation [60,61]. Fisher’s Least Significant Difference (LSD) tests as well as 95% upper and lower confidence intervals (CIs) at pre-planned contrasts of interest were used to assess pairwise comparisons of means and post hoc tests. The probability of type I statistical error was 0.05 or less. Statistical trends were identified when *p*-values ranged between 0.05 and 0.10. Effect size was analyzed using Partial Eta squared (η_p_^2^) where values of 0.01 represented a small effect, 0.06 represented a medium effect, and 0.14 represented a large effect size [62]. Clinical significance was examined by assessing changes from the baseline with 95% CI [56]. Data are means ± standard deviations (SDs) or mean percent changes from the baseline (mean change [LL, UL]). Numerical missing data were replaced using the series means [63], while ordinal data from surveys were replaced using the most frequent response or value method [64].

## 4. Results

### 4.1. Participant Demographics

Table S1 shows demographic data and baseline clinical markers for the individuals who participated in this study. Participants were 56.0 ± 9.0 years, 164.4 ± 14 cm, 86.5 ± 20.9 kg, and 31.0 ± 7.5 kg/m^2^. Multivariate GLM analysis revealed no significant sex (*p* = 0.456, η_p_^2^ = 0.986), group (*p* = 0.437, η_p_^2^ = 0.987), or group × sex (*p* = 0.587, η_p_^2^ = 0.974) within-subject effects. No significant sex differences were observed among the baseline variables, with the exception that red blood cells (−0.38 K/µL [−0.74, −0.02], *p* = 0.039) were lower in women while basophils (−0.24% [−0.46, −0.03], *p* = 0.029), alkaline phosphatase (−21.8 U/L [−43.0, −0.5], *p* = 0.045), and IL−6 (−1.42 pg/mL [−2.4, −0.5], *p* = 0.005) concentrations were lower in males at the baseline. No baseline group × sex effects were observed among groups. Given these findings and the fact that sex differences from ginger supplementation have generally not been reported in the literature [20,37], we only report group, time, and group × time effects in the remaining analyses.

### 4.2. Exercise Stimulus

Table S2 presents the amount of weight lifted and the total lifting volume from the weighted knee bend exercise per set during the study. Participants lifted about 50 lbs. for each set of ten repetitions. Multivariate Wilk’s Lambda showed no significant time (*p* = 0.113, η_p_^2^ = 0.089) or group × time (*p* = 0.560, η_p_^2^ = 0.043) effects.

### 4.3. Primary Outcomes

#### 4.3.1. Ratings of Muscle Pain

Participant ratings of pain in response to the application of pressure at three locations on the quadriceps muscle are shown in Table S3. Chi-squared analysis revealed that participants tended to rate pain as more severe at the vastus medialis site after two days of recovery from session one testing (day 2: χ^2^ *p* = 0.087). However, no other differences were observed between groups at other data points.

#### 4.3.2. Osteoarthritis Severity Indices

**Figure 3:**
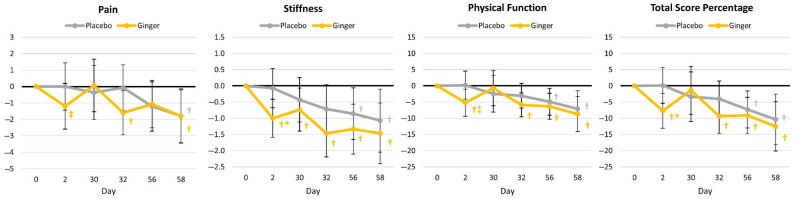
Western Ontario and McMaster University Osteoarthritis Index (WOMAC™) pain, stiffness, physical function, and total scores. † represents *p* < 0.05 (‡ *p* > 0.05 to *p* < 0.10) difference from baseline values. * represents *p* < 0.05 (⁑ represents *p* > 0.05 to *p* < 0.10) difference between groups.

The chi-squared analysis of individual items from the WOMAC Osteoarthritis Index questionnaire is presented in Table S4, while the GLM analysis of total scores is shown in Table S5. Participants in the ginger group reported less pain from sitting two days after baseline testing (χ^2^ *p* = 0.002), while nocturnal pain (day 32: χ^2^ *p* = 0.075, day 58: (χ^2^ *p* = 0.097), weight-bearing pain (day 32: χ^2^ *p* = 0.058), physical functioning of performing heavy duties (day 32: χ^2^ *p* = 0.100), and physical functioning of performing light duties (day 2: χ^2^ *p* = 0.064) tended to have less severe ratings with ginger supplementation. Conversely, the placebo group tended to rate less pain from bending to the floor (day 58: χ^2^ *p* = 0.057). A significant overall time (*p* < 0.001, η_p_^2^ = 0.100), but not group × time (*p* = 0.690, η_p_^2^ = 0.029) effect was observed in WOMAC pain, stiffness, physical function, and total scores. Univariate analysis indicated that time effects were observed in each of these markers with no significant differences observed between groups. However, when changes from baseline values with 95% CIs were examined (Figure 3), participants in the ginger group experienced significant reductions in ratings of pain, stiffness, physical function, and total score values, particularly in 48 h follow-up measures.

#### 4.3.3. Lequesne Index of Severity of Hip Osteoarthritis

**Figure 4:**
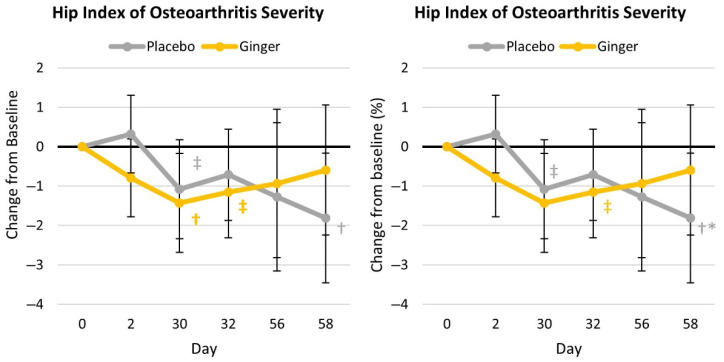
Lequesne Index of Severity of Hip Osteoarthritis total scores. † represents *p* < 0.05 (‡ *p* > 0.05 to *p* < 0.10) difference from the baseline values. * represents *p* < 0.05 difference between groups.

Table S6 presents results from the Lequesne Osteoarthritis Index ratings. The chi-squared analysis revealed that participants in the ginger group reported less pain from sitting (day 2: χ^2^ *p* = 0.020); discomfort during nighttime bed rest (day 2: χ^2^ *p* = 0.065; day 32: χ^2^ *p* = 0.032); and when going up and down stairs (day 32: χ^2^ *p* = 0.097). Total scores are shown in Table S7. Ratings of hip osteoarthritis severity scores tended to decrease over time (*p* = 0.066, η_p_^2^ = 0.079) with no group × time effects observed (*p* = 0.275, η_p_^2^ = 0.045). Total score changes from the baseline values are shown in Figure 4. Ratings of hip osteoarthritis severity significantly decreased from the baseline after 30 days in the ginger group and after 58 days in the placebo group, with no significant difference observed between groups except when expressed as a percentage change from the baseline at day 58.

#### 4.3.4. Inflammatory Markers

**Figure 5:**
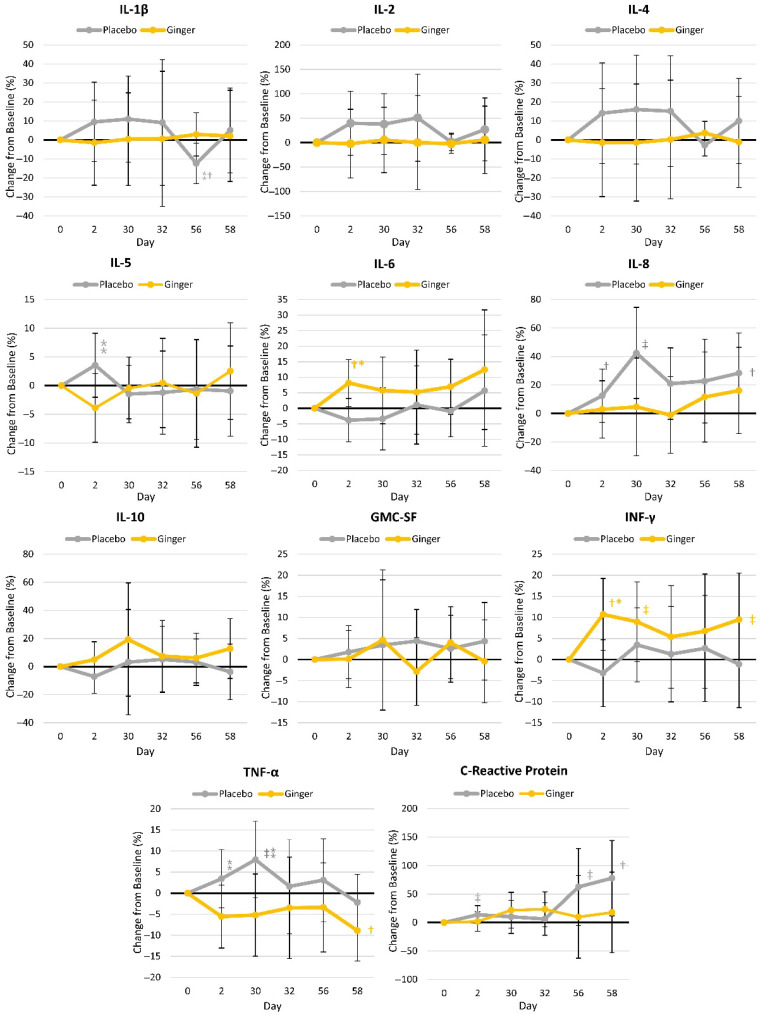
Inflammatory markers. IL = Interleukin, GMC-SF = Granulocyte-Macrophage Colony-Stimulating Factor, INF = interferon, TNF = Tumor Necrosis Factor. † represents *p* < 0.05 (‡ *p* > 0.05 to *p* < 0.10) difference from the baseline values. * represents *p* < 0.05 (⁑ represents *p* > 0.05 to *p* < 0.10) difference between groups.

Table S8 presents the inflammatory and anti-inflammatory markers evaluated in this study. Multivariate Wilk’s Lambda showed no significant time (*p* = 0.998, η_p_^2^ = 0.045) or group × time (*p* = 0.448, η_p_^2^ = 0.084) within-subject effects. Univariate analysis revealed that the ginger treatment had lower group mean IL-1β (*p* = 0.050, η_p_^2^ = 0.140), TNF-α (*p* = 0.064, η_p_^2^ = 0.126), GMC-SF (*p* = 0.001, η_p_^2^ = 0.346), and IL-5 (*p* = 0.047, η_p_^2^ = 0.144) concentrations. However, no significant univariate time or group × time effects were observed. Figure 5 shows percent changes from the baseline in inflammatory markers. Significant time effects and/or differences between groups were seen in IL-β, IL-5, IL-6, IL-8, INF-γ, TNF-α, and high-sensitivity C-Reactive Protein at various points during the study.

### 4.4. Secondary Outcomes

#### 4.4.1. Flexibility and Range of Motion

**Figure 6:**
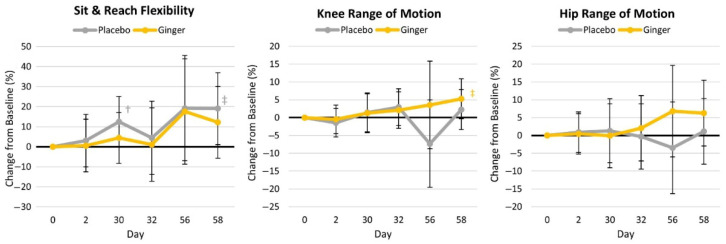
Flexibility and range of motion changes from the baseline. † represents *p* < 0.05 (‡ *p* > 0.05 to *p* < 0.10) difference from the baseline values.

Table S9 presents the results for low back flexibility and the range of motion of the knees and hips. Multivariate GLM analysis revealed no significant time (*p* = 0.244, η_p_^2^ = 0.043) or group × time (*p* = 0.715, η_p_^2^ = 0.027) effects. Univariate analysis revealed that sit-and-reach values tended to be higher in the placebo group overall (*p* = 0.096, η_p_^2^ = 0.096), as well as after 30 days of supplementation (0.044 cm [0.14, 10.3], *p* = 0.044), with both groups increasing their flexibility over time (*p* = 0.051, η_p_^2^ = 0.084). However, no significant group × time interaction effects were observed (*p* = 0.798, η_p_^2^ = 0.013). Knee range of motion tended to be higher in the ginger group (*p* = 0.063, η_p_^2^ = 0.118), with values tending to be higher in the ginger group (day 56: 20.8° [−1.6, 43.2], *p* = 0.068; day 58: 9.4° [−1.9, 20.7], *p* = 0.100). No group, time, group × time, or pairwise comparison differences were observed in hip range of motion. Percent changes from the baseline values are presented in Figure 6.

#### 4.4.2. Quality of Life

Table S10 shows a chi-squared analysis of the SF-36 QOL inventory. Results revealed significant differences between groups on various testing days in their ratings of health compared to one year ago, including reducing the volume of time allocated to work and other activities, experiencing bodily pain over the last four weeks, feeling full of life, and anticipating their health to worsen. Perceptions tended to differ between groups when asked to rate whether their health limits the carrying of groceries, results in accomplishing less than they would like, limits the kind of work/activities they can partake in, cuts down on time spent on work or other activities, and feeling nervous, happy, tired, healthy as anybody, and that their health was excellent. However, while participants taking ginger reported less bodily pain and perceived greater happiness, other responses were mixed or not as positive as those in the placebo group.

#### 4.4.3. Blood Markers of Health

**Figure 7:**
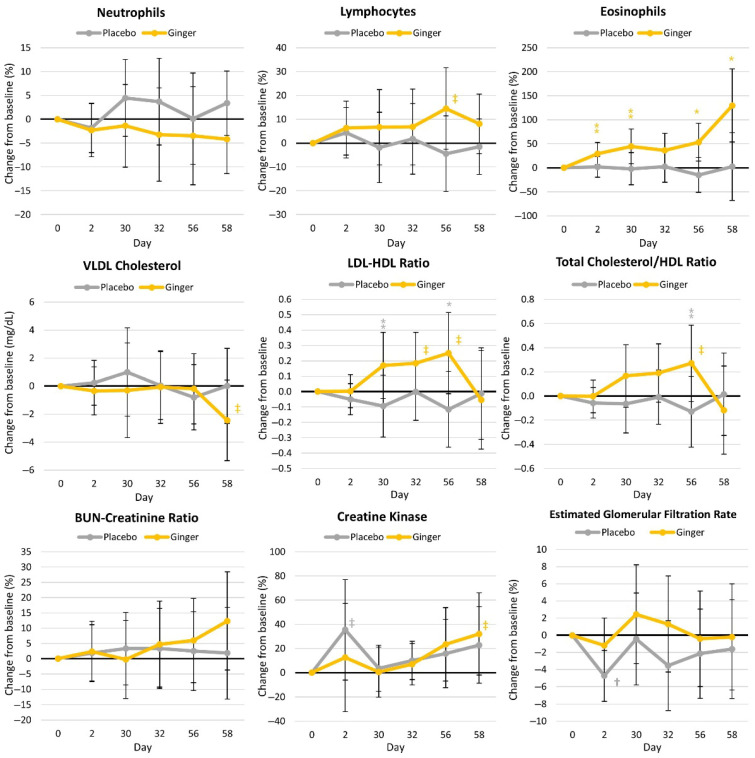
Selected blood markers of health. VLDL = very-low-density lipoprotein, LDL = low-density lipoproteins, HDL = high-density lipoproteins, and BUN = blood urea nitrogen. † represents *p* < 0.05 (‡ = *p* > 0.05 to *p* < 0.10) difference from the baseline values. * represents *p* < 0.05 (⁑ = *p* > 0.05 to *p* < 0.10) difference between groups.

Tables S11–S14 present cell blood count (CBC), serum lipids, markers of renal function and electrolytes, and protein and bone markers, respectively. No significant multivariate time effects were seen in cell blood count (*p* = 0.485, η_p_^2^ = 0.084). However, CBC values tended to interact over time (*p* = 0.052, η_p_^2^ = 0.135). Univariate analysis revealed that eosinophil values tended to increase over time in the ginger group (*p* = 0.009, η_p_^2^ = 0.130), with significantly higher eosinophil levels observed after 58 days. Additionally, some group differences were observed in hemoglobin, mean corpuscular volume, mean corpuscular hemoglobin concentration, and red blood cell distribution width, with values lower in the ginger group. However, no significant time or group × time effects were observed in these markers. No multivariate time (*p* = 0.651, η_p_^2^ = 0.055) or interaction effects (*p* = 0.291, η_p_^2^ = 0.067) or univariate group, time, or group × time effects were observed in blood lipid levels (see Table S12). The low-density lipoprotein (LDL) to high-density lipoprotein (HDL) ratio and total cholesterol–HDL ratio tended to increase over time in the ginger group after 56 days of supplementation, with differences observed between groups in cholesterol (22.1 mg/dL [−4.3, 48.3], *p* = 0.096), LDL cholesterol (25.3 mg/dL [0.1, 50.6], *p* = 0.049), non-HDL cholesterol (26.5 mg/dL [−1.1, 53.7], *p* = 0.060), and the LDL–HDL ratio (0.64 [−0.05, 1.3], *p* = 0.068). However, no significant differences were observed between groups when expressed as means and percentage changes from the baseline. A significant multivariate time (*p* = 0.037, η_p_^2^ = 0.103) effect was observed in renal and electrolyte markers (see Table S13). However, no significant interaction (*p* = 0.496, η_p_^2^ = 0.075) or univariate group, time, or interaction effects were observed. Finally, Table S14 shows that no overall time effect (*p* = 0.994, η_p_^2^ = 0.027) or interaction effect (*p* = 0.335, η_p_^2^ = 0.057) was observed from multivariate analysis of protein-related markers. Group effects were seen in the albumin–globulin ratio, which was lower in the ginger group. However, no other group, time, or group × time effects were observed. Pairwise comparisons revealed that fasting blood glucose values were lower in the ginger group after 56 (−11.5 mg/dL [−25.1, 2.0], *p* = 0.092) and 58 (−13.8 mg/dL [−26.1, −1.6], *p* = 0.028) days. However, no differences were observed between groups when expressed as changes from the baseline or percent changes from the baseline. All values were within normal values for healthy individuals. Figure 7 displays mean percentage changes from the baseline with 95% CIs in whole blood and serum markers of interest.

#### 4.4.4. Resting Hemodynamics

**Figure 8:**
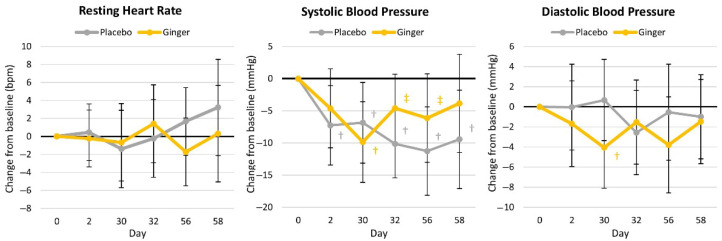
Changes in resting heart rate and blood pressure. † represents *p* < 0.05 (‡ = *p* > 0.05 to *p* < 0.10) difference from the baseline values.

Table S15 presents resting heart rate and blood pressure responses. Multivariate analysis revealed a significant time effect (*p* = 0.046, η_p_^2^ = 0.058) but no significant interaction effects (*p* = 0.209, η_p_^2^ = 0.044). The resting heart rate tended to be higher in the ginger group (*p* = 0.067, η_p_^2^ = 0.115), with no interaction effect observed (*p* = 0.456, η_p_^2^ = 0.031). Systolic blood pressure decreased over time (*p* = 0.005, η_p_^2^ = 0.124) with no interaction effects (*p* = 0.310, η_p_^2^ = 0.042). No group, time, or group × time effects were seen in diastolic blood pressure. Mean changes from the baseline with 95% CIs are shown in Figure 8.

#### 4.4.5. Analgesic Use and Side Effects

Table 1 shows the total number of individuals in each group who used rescue doses of OTC analgesics (e.g., ibuprofen, acetaminophen, naproxen, and aspirin) to manage pain. A total of 73.3% of participants in the placebo group reported using rescue doses of analgesics at least once during the study. In comparison, 46.7% of participants in the ginger group reported taking analgesics. However, the chi-squared analysis did not reveal significant differences in the number of participants reporting the use of rescue doses of analgesics during baseline testing (χ^2^ *p* = 0.195), after 4 weeks (χ^2^ *p* = 0.232), and after 8 weeks of supplementation (χ^2^ *p* = 0.713).

Table S16 presents responses to questions regarding the frequency and severity of side effects. Although participants typically rated side effects as infrequent (1–2 times/week) and of minimal to slight severity, there was some evidence that participants in the ginger group reported more frequent and severe headaches, heart palpitations, and nervousness.

## 5. Discussion

Ginger contains gingerols, shagols, paradols, and zingerone [1,4,5,6], which have been reported to possess anti-inflammatory [18,26,29,65], antioxidant [18,65], immuno-modulatory [8,14,66], and pain suppression [17,18,19,20] effects by inhibiting macrophage and neutrophil activation, decreasing pro-inflammatory cytokines that influence the COX inflammatory pathway [1,2], and inhibiting TRPV1 pain-sensitive receptors. Theoretically, ginger could serve as a natural alternative to anti-inflammatory medications. However, most sources of ginger only provide 1–2% gingerols, requiring the ingestion of 1–3 g/d of powdered ginger daily (e.g., 1–2 × 500 mg capsules, two–three times per day) to provide therapeutic levels of gingerol, which makes compliance difficult. Consequently, there has been interest in developing ginger extract powder with higher concentrations of specific gingerols and shogaols. This study investigated whether the ingestion of 125 mg/d of a ginger extract, providing 12.5 mg of gingerols, affects perceptions of pain, functional capacity, markers of inflammation, and related variables in individuals experiencing mild to moderate joint pain. The primary findings indicated that the source and dosage of ginger used in this study lessened some perceptions of pain, improved characteristics of perceived functional capacity, and reduced several markers of inflammation. In addition, this investigation displayed evidence that ginger supplementation influenced antioxidant status and general markers of immunity. The following discussion considers these findings in relation to the existing literature, outlines the study’s limitations, and provides recommendations for future research needs.

### 5.1. Primary Outcomes

#### 5.1.1. Perceptions of Pain and Functional Capacity

The primary reason individuals with mild to moderate muscle and joint pain take prescription medications, OTC analgesics, and/or natural alternatives to medications is to manage pain, improve their capacity to carry out functional activities, and reduce perceptions of pain following physical activity. Ginger has been reported to reduce perceptions of pain among individuals with mild to moderate muscle and joint pain. For example, Altman et al. [34] reported that the dietary supplementation of ginger (providing about 30 mg/d of gingerols) for 6 weeks reduced perceptions of pain upon standing and walking in osteoarthritis patients. Wigler and coworkers [67] reported that ginger supplementation (providing 40 mg/d of gingerols) for 24 weeks improved perceptions of pain and feelings about physical limitations. Zakeri and colleagues [68] found that ginger supplementation (providing 12.3 mg/d of gingerols) for 6 weeks reduced perceptions of pain and morning stiffness. Additionally, Black et al. [9,69] reported that the ingestion of ginger (providing 8.6 and 19 mg/d of gingerols) reduced perceptions of pain after exercise. In response to exercise, Mashhadi and coinvestigators [70] reported that taekwondo athletes experienced lower perceptions of muscle soreness during 8 weeks of training when supplementing their diet with ginger (providing 30 mg/d of gingerols). Similar findings were reported by Hoseinzadeh et al. [71] and Dominguez-Balmaseda and associates [72] when supplementing the diet with ginger (providing 60 mg/d and 27 mg/d of gingerols, respectively) for recovery from intense exercise. In 2015, Bartels et al. [73] conducted a meta-analysis and found that ginger supplementation (500–1000 mg/d for 3–12 weeks) in osteoarthritic patients significantly reduced perceptions of pain and disability comparable or better than other oral analgesics [74,75]. In the current investigation, participants in the supplemental ginger group tended to rate less pain in the vastus medialis from the application of standard pressure, and reported lower ratings of pain, stiffness, physical function limitations, and total score values (meaning less limitations) on the Western Ontario and McMaster Universities Osteoarthritis Index (WOMAC) Osteoarthritis Global Index questionnaire, particularly in post-exercise follow-up sessions. There was also evidence that participants in the ginger group reported less discomfort while sleeping at night, going up and down stairs, as well as less hip osteoarthritis severity over time in the Lequesne Functional Index Questionnaire. These findings support contentions that the dietary supplementation of this concentrated source of ginger (containing 12.5 mg/d of gingerols) for 8 weeks may lessen perceptions of pain and improve perceptions about functional capacity in individuals experiencing mild to moderate joint pain and thereby serve as a natural analgesic.

#### 5.1.2. Markers of Inflammation

A primary mechanism by which ginger is purported to reduce pain perception is by reducing inflammation. Prior in vitro studies indicated that cells incubated with gingerols suppressed IL-6 [7,11], IL-8 production [7], and TNF-α [11] levels. Rodent studies have reported that gingerol administration significantly reduces the inflammatory markers IL-1β [7,11], IL-6 [11], IL-12 [11], IL-17 [11], TNF-α [7,10,76], and NF-κβ [10,12,76]. Human studies have shown that ginger supplementation (containing 20–60 mg/d of gingerols) significantly decreased fasting IL-1β [26], IL-6 [29,77], TNF-α [26,29,65,77], C-Reactive Protein [77,78,79], and PGE_2_ [79]. In contrast, other studies report no effects on TNF-α [29,79] or C-Reactive Protein [27,80] in clinical populations. In terms of exercise, several studies have reported that acute dietary supplementation with ginger has no effect on creatine kinase [25,71,72] or prostaglandin E_2_ (PGE_2_) levels [9]. However, ginger supplementation during exercise training has been shown to reduce IL-1β [59], IL-6 [59,64], TNF-α [59], and C-Reactive Protein [72]. The present study evaluated the impact of 8 weeks of supplemental ginger (125 mg/d providing 12.5 mg/d of gingerols) on fasting inflammatory markers as well as recovery from resistance exercise designed to promote muscle soreness and inflammation in untrained participants with mild to moderate muscle and joint pain. Consequently, while we evaluated the effects of an exercise stimulus during the supplementation period, we did not evaluate the effects of ginger during training. The results revealed evidence that ginger supplementation attenuated increases in IL-5 (a cytokine that stimulates B-cell growth and immunoglobulin IgA production), IL-8 (a pro-inflammatory cytokine that helps recruit leukocytes to sites of inflammation), TNF-α (a pro-inflammatory cytokine involved in the acute phase response), and C-reactive protein (an acute inflammatory protein). However, IL-6 (a pro- and anti-inflammatory cytokine involved in the acute-phase inflammatory response and B-cell growth) and IFN-γ (a pro-inflammatory cytokine that promotes T-cell helper immune responses) were significantly higher after the baseline exercise stimulus. While these data may seem contradictory, higher IL-6 concentrations may have influenced the reduction in TNF-α over time. Additionally, IL-6 has immunomodulatory effects on humoral immunity, affecting B-cell function. Given that we observed increases in whole-blood lymphocytes, eosinophils, and basophils, the higher IL-6 may have reflected an immunomodulating effect. Nevertheless, the results suggest that ginger supplementation reduces markers of inflammation while promoting certain immunoenhancing effects. These findings support reports that gingerols possess anti-inflammatory and immunomodulating effects.

### 5.2. Secondary Outcomes

We evaluated several secondary variables to further explore the potential effects of ginger supplementation on functional capacity and QOL. Similarly to Haghighi et al. [74] and Bliddal and associates [75], ginger supplementation did not significantly improve low back flexibility or knee and hip ROM. Nevertheless, consistent with the results of the osteoarthritis questionnaires, participants taking ginger reported less bodily pain and more often feeling full of life in the SF-36 QOL inventory. Other responses were not significantly different between groups or, in some instances, more positive among participants in the placebo group. Comparatively, Niempoog et al. [81] reported that ginger supplementation (1 g/d providing an estimated 10–20 mg/d gingerols for 8 weeks) did not significantly affect measures of QOL in osteoarthritis patients. The dietary supplementation of ginger has also been shown to improve fasting glucose [79], HbA1C [79], insulin [79], and the homeostatic model of insulin resistance, HOMA_IR_ [79]. In the present study, glucose values after 56 and 58 days of supplementation were lower in the ginger group when expressed in absolute terms (mg/dL), but not when comparing changes from the baseline. Several studies have found that ginger supplementation (1.6–3.0 g/d providing 16–60 mg/d of gingerols) lowered triglycerides [79], total cholesterol [79,80], HDL [80], and LDL [80]. In this study, blood lipids were relatively low throughout the study, particularly in the placebo group. Nevertheless, the LDL–HDL ratio and total cholesterol–HDL ratio tended to increase over time in the ginger group after 56 days of supplementation, with a difference observed between groups in cholesterol (*p* = 0.096), LDL cholesterol (*p* = 0.049), non-HDL cholesterol (*p* = 0.060), and the LDL–HDL ratio (*p* = 0.068). However, no significant differences were observed between groups when expressed as means and percentage changes from the baseline. These findings do not support contentions that ginger supplementation improved blood lipid profiles. Finally, creatine kinase (CK) increases after intense exercise, typically peaking at about 24 h. It is often used to assess muscle damage. Multiple investigations have observed no effect of ginger supplementation on creatine kinase (CK) levels after exercise [7,25,71]. Results from this study support those findings.

### 5.3. Safety and Side Effects

Ginger supplementation has been reported to be well tolerated when consuming 1–3 g/d for 4–48 weeks, providing 10–60 mg/d of gingerols [17,73]. In the current investigation, we evaluated the effects of dietary supplementation with 125 mg/d of ginger (providing 19 mg/d of gingerols) for 8 weeks. A comprehensive panel of whole blood and serum and responses to a side effect frequency and severity questionnaire were used to examine clinical safety. While some changes over time were observed within and between groups in pair-wise comparisons, no significant interaction effects were observed in markers of renal function, electrolytes, protein and bone markers, or resting blood pressure and heart rate. Most differences between groups were not observed when evaluating mean and percentage changes from the baseline. Some participants who took ginger reported more frequent and/or severe headaches, heart palpitations, and/or nervousness. However, the incidence was generally low (none to one–two times/week) with minimal to slight severity, and typically involved only a few participants changing ratings from their baseline responses. No differences were observed in ratings of the frequency or severity of side effects. Finally, while not statistically significant, there was evidence that participants taking ginger were less dependent on taking rescue doses of OTC analgesics. These findings support contentions that ginger supplementation appears to be well tolerated. However, it should be noted that most studies conducted on ginger supplementation have been less than 12 weeks in duration [20,37].

### 5.4. Strengths, Limitations, and Future Directions

The strengths of this study were that this study (1.) evaluated the safety and efficacy of supplementing the diet with a low-dose source of ginger (125 mg/d) that provided similar amounts of gingerol than higher-dose sources of ginger (i.e., 1–2 g/d) in individuals with perceptions of low to moderate muscle and joint pain; (2.) included a relative resistance exercise stimulus to assess the effectiveness of supplemental ginger on muscle pain and recovery; (3.) evaluated perceptions of pain to a standardized pressure with functional capacity questionnaires; (4.) assessed a broad array of pro- and anti-inflammatory cytokines to assess inflammation and immune responses to exercise; (5.) involved a comprehensive statistical analysis to assess statistical outcomes and the clinical significance of findings; and (6.) evaluated markers of health, safety, and side effects. The investigation was limited by its sample size, the dosage and length of the intervention (125 mg/d, providing 12.5 mg/dL of gingerols for 8 weeks), the exercise protocol employed, only evaluating recovery from the exercise stimulus after 48 h which may have missed some potential effects after 24 and/or 72 h of recovery, the population studied, and participant compliance. Future research should evaluate whether ingesting this source of ginger more frequently (e.g., twice a day), for longer periods of time, prior to exercise, and in healthy and active individuals may experience benefits; how the chronic use of this source of ginger may impact the use of OTC analgesics in individuals with and without mild to moderate joint and muscle pain; and the long-term safety of ginger supplementation on clinical health markers, including liver function.

## 6. Conclusions

Low-dose ginger supplementation (125 mg/d providing 12.5 mg/d of gingerols) lessened some perceptions of pain, improved perceptions related to functional capacity, and reduced several markers of inflammation in individuals who experience mild to moderate joint and muscle pain. These findings support prior reports that ginger extracts and gingerols contain anti-inflammatory and immunomodulating effects that may help reduce perceptions of pain in individuals with chronic muscle and knee pain. The results also offer promise that smaller quantities of ginger with higher contents of gingerols may serve as an effective source of ginger for individuals with chronic joint and muscle pain to help manage pain. The results also support prior reports that ginger supplementation is well tolerated. In terms of practical recommendations, individuals with chronic joint and muscle pain, as well as clinicians treating these types of patients, may want to consider ginger as a natural alternative and/or adjunctive dietary supplement to OTC and/or prescription pain medications. Finally, while studies have consistently reported safety for up to 12 weeks of supplementation, additional research should evaluate the safety of longer periods of supplementation on clinical markers of health.

## Figures and Tables

**Table 1:** Number of participants using over-the-counter analgesic medications.

Group	Week 0	χ^2^ *p*-Level	Week 4	χ^2^ *p*-Level	Week 8	χ^2^ *p*-Level	Total Used Analgesics	Percent Used Analgesics
Placebo	5	0.195	6	0.232	7	0.713	11	73.3%
Ginger	2		3		6		7	46.7%

## Data Availability

Data and statistical analyses are available for non-commercial scientific inquiry and/or educational use if requested and their use does not violate IRB restrictions and/or research agreement terms. This is to ensure the privacy of participant data and ensure that sharing data does not violate research agreements or state law concerning intellectual property.

## Supplementary Materials

The following supporting information can be downloaded at: https://www.mdpi.com/article/10.3390/nu17142365/s1. Table S1: Descriptive demographic and clinical data, Table S2: Weighted knee bend weight lifted and volume, Table S3: Ratings of pain in response to standardized pressure, Table S4: WOMAC™ Osteoarthritis Index results, Table S5: WOMAC Osteoarthritis Index pain, stiffness, physical function, and total scores, Table S6: Lequesne Index of Severity of Hip Osteoarthritis results, Table S7: Lequesne Index of Severity of Hip Osteoporosis total score, Table S8: Cytokine analysis results, Table S9: Low back flexibility and knee and hip range of motion, Table S10: Quality of life ratings, Table S11: Complete blood count results, Table S12: Serum lipid results, Table S13: Serum renal function and electrolyte markers, Table S14: Serum protein and bone markers, Table S15: Resting heart rate and blood pressure, Table S16a: Frequency of side effects, Table S16b: Severity of side effects.

## Abbreviations

The following abbreviations are used in this manuscript:

Akt	Protein Kinase B
ANOVA	Analysis of variance
CI	Confidence interval
CONSORT	Consolidated Standards of Reporting Trials
COX-2	Cyclooxygenase enzyme-2 isoenzyme
CK	Creatine kinase
DMARDS	Disease-modifying antirheumatic drugs
GLM	General linear model
GMC-SF	Granulocyte-macrophage colony-stimulating factor
HDL	High-density lipoprotein
HOMA_IR_	Homeostatic model assessment for insulin resistance
IFN-γ	Interferon-γ
IL	Interleukin
LDL	Low-density lipoproteins
LSD	Least Significant Difference
NF-κβ	Nuclear Factor-kappa β
OTC	Over the counter
NSAIDS	Nonsteroidal anti-inflammatory drugs
QOL	Quality of life
ROM	Range of motion
SD	Standard deviation
SF-36	Short Form Health Survey, 36-item
TNF-α	Tumor Necrosis Factor-α
TRPV1	Vanilloid subtype 1 pain-sensitive receptor
VAS	Visual analog scale
VLDL	Very-low-density lipoprotein
WOMAC	Western Ontario and McMaster Universities Osteoarthritis Index
